# Effect of isoform-specific HIF-1α and HIF-2α antisense oligonucleotides on tumorigenesis, inflammation and fibrosis in a hepatocellular carcinoma mouse model

**DOI:** 10.18632/oncotarget.27830

**Published:** 2020-12-01

**Authors:** Bart Vanderborght, Kevin De Muynck, Sander Lefere, Anja Geerts, Helena Degroote, Xavier Verhelst, Hans Van Vlierberghe, Lindsey Devisscher

**Affiliations:** ^1^Department of Internal Medicine and Pediatrics, Department of Gastroenterology and Hepatology, Hepatology Research Unit, Ghent University, Ghent, Belgium; ^2^Department of Basic and Applied Medical Sciences, Gut-Liver Immunopharmacology Unit, Ghent University, Ghent, Belgium

**Keywords:** hepatocellular carcinoma, hypoxia-inducible factor, antisense oligonucleotides, tumor microenvironment, experimental mouse model

## Abstract

Hepatocellular carcinoma (HCC) is one of the leading causes of cancer-related death worldwide. For advanced HCC, there is still an unmet need for more effective therapeutic strategies. HCC is typically associated with hypoxia and the hypoxia-inducible factor (HIF) regulatory pathway plays an important role in HCC development and progression. Therefore, we investigated the therapeutic potential of isoform-specific HIF-1α and HIF-2α antisense oligonucleotides (ASOs), along with their effect on the inflammatory and fibrotic component of the tumor microenvironment (TME), in an experimental HCC mouse model. Based on its efficacy and safety, a dosage regimen of 20 mg/kg intraperitoneal injection of HIFα ASO twice per week was selected for further investigation in a preventive and therapeutic setting in a N,N-diethylnitrous amide (DEN)-induced HCC mouse model. DEN administration resulted in 100% tumor formation and HIFα ASO administration led to effective and selective hepatic downregulation of its target genes. HIFα ASO treatment had no effect on tumor numbers, but even enhanced the increased hepatic expression of HCC tumor markers, α-fetoprotein and glypican-3, compared to scrambled control ASO treatment in HCC mice. Especially HIF-1α ASO treatment resulted in an enhanced increase of monocytes and monocyte-derived macrophages in the liver and an enhanced hepatic upregulation of inflammatory markers. Both HIFα ASOs aggravated liver fibrosis in HCC mice compared to scrambled ASO treatment. The observed effects of our dosing regimen for HIF-1α and HIF-2α ASO treatment in the DEN-induced HCC mouse model discourage the use of HIFα isoforms as targets for the treatment of HCC.

## INTRODUCTION

Hepatocellular carcinoma (HCC) represents the majority of primary liver cancer cases and is currently the fourth most common cause of cancer-related death worldwide [1–2]. HCC usually occurs in a background of chronic liver disease, mainly caused by viral hepatitis, chronic alcohol abuse or non-alcoholic fatty liver disease (NAFLD) [1, 3]. This environment of repetitive hepatic damage and genomic instability contributes to the broad array of genetic and epigenetic alterations by which the heterogeneous molecular pathogenesis of this malignancy is characterized [4]. A frustrating discrepancy exists between the stage at which HCC is commonly first diagnosed and the stage at which curative treatment options are currently available [3]. HCC is an aggressive cancer and is often diagnosed at an advanced stage, while possible curative interventions, including ablation, resection and liver transplantation, are only effective at an early disease stage [3, 5]. For advanced HCC, several systemic therapies with minor survival benefits and considerable adverse events are available in the form of multikinase inhibitors and immune checkpoint inhibitors. Currently, two oral tyrosine kinase inhibitors are approved as first-line treatment of advanced HCC, namely sorafenib and lenvatinib [1, 2]. For second-line treatment, the currently approved options are the multitargeted tyrosine kinase inhibitors regorafenib and cabozantinib, and the human monoclonal vascular endothelial growth factor receptor (VEGFR)2-targeting antibody ramucirumab, which all yield only limited clinical benefits [2]. In addition to these targeted therapies, immune-based therapies are, due to their relatively higher response rates, emerging as promising treatment options for advanced HCC. Currently, the anti-programmed cell death protein 1 (PD-1) antibodies nivolumab and pembrolizumab are the only immune checkpoint inhibitors approved as second-line therapy for advanced HCC following failure of sorafenib [1, 6]. The clinical benefit of other immune checkpoint-targeting therapies, including the cytotoxic T-lymphocyte-associated protein 4 (CTLA-4) inhibitor tremelimumab, and the synergistic effect of combination therapies of kinase inhibitors, immune checkpoint inhibitors and/or locoregional therapies are being extensively investigated in clinical trials [6]. However, despite the emergence of immunotherapy in the treatment landscape of HCC, there is still an unmet need for more effective therapeutic strategies [7].

In order to sustain their tumorigenicity and proliferative behavior, HCC cells have the ability to metabolically adapt to a nutrient-deprived microenvironment [8–9]. Indeed, cancer cells are able to reprogram their energy metabolism towards aerobic glycolysis, a phenomenon called the Warburg effect [10–12]. Due to the oxygen-consuming hypermetabolism of the rapidly proliferating tumor cells, HCC is typically associated with hypoxia in the intratumoral regions [13–14]. This hypoxic microenvironment promotes tumor aggressiveness and therapeutic resistance primarily through activation of the hypoxia-inducible factor (HIF) regulatory pathway [14, 15]. This hypoxia-responsive pathway consists of α-subunits (HIFα, including HIF-1α, HIF-2α/EPAS1 and HIF-3α) and β-subunits (HIFβ, including HIF-1β/ARNT1, ARNT2 and ARNT3) [15]. Under normoxia, the HIFα subunit is hydroxylated at two proline residues by prolyl hydroxylase domain-containing protein (PHD), and subsequently ubiquitinated by von Hippel-Lindau tumor suppressor protein (pVHL) and degraded by the 26S proteasome. Additionally, factor inhibiting HIF (FIH) mediates asparaginyl hydroxylation of HIFα, thereby inhibiting its interaction with transcriptional coactivators CREB-binding protein (CBP) and p300. Both PHD and FIH are oxygen-dependent enzymes, which implies that they are inactive under hypoxic conditions. Therefore, in hypoxia-associated cancers, including HCC, the HIFα subunit is stabilized, leading to its nuclear translocation. After dimerization with the constitutively expressed HIFβ subunit, and interaction with transcriptional activators CBP and p300, the resultant heterodimer acts as a transcription factor, upregulating the expression of a large number of hypoxia-responsive target genes by binding to the hypoxia response element (HRE) in their promoter region [13, 15]. These genetic targets comprise multiple cancer hallmark-implicated genes, including genes involved in 1) angiogenesis, such as vascular endothelial growth factor (VEGF), erythropoietin (EPO) and platelet-derived growth factor (PDGF); 2) metabolism, such as glucose transporter 1 (GLUT1), glyceraldehyde 3-phosphate dehydrogenase (GAPDH) and phosphoglycerate kinase 1 (PGK1); 3) proliferation, such as insulin-like growth factor 2 (IGF-2) and transforming growth factor (TGF)-α; and 4) invasion and metastasis, such as lysyl oxidase (LOX) and several matrix metalloproteinases (MMPs). Thus, in HCC and other hypoxia-associated tumors, the HIF pathway has a key role in shaping the tumor microenvironment (TME) [13, 15–18]. Consequently, targeting hypoxia and, more specifically, the HIF pathway appears to be a plausible therapeutic strategy for the treatment of HCC [13, 15]. To date, in addition to downstream HIF signaling pathway-targeting strategies, including the approved VEGFR-targeting drugs sorafenib, lenvatinib, regorafenib, cabozantinib and ramucirumab, an array of HIF-targeting compounds have been identified and investigated in preclinical studies and clinical trials [13, 15, 19–20]. A substantial part of these HIF-targeting compounds are inhibitors of HIF messenger ribonucleic acid (mRNA) or protein expression. These include compounds that 1) target the phosphoinositide 3-kinase/protein kinase B/mammalian target of rapamycin (PI3K/AKT/mTOR) pathway (regulates HIFα mRNA translation), 2) inhibit topoisomerase 1 (regulates HIFα mRNA translation), 3) directly target HIF-1α mRNA expression (synthetic HIF-1α antisense oligonucleotides), 4) disrupt microtubules (orchestrate HIFα mRNA translation), 5) inhibit heat shock protein 90 (Hsp90), which induces proteasomal degradation of HIFα, 6) inhibit histone deacetylase (HDAC), which inhibits nuclear translocation of HIFα, or 7) promote iron-regulatory protein 1/iron-responsive element (IRP1/IRE) interaction (HIF-2α translational inhibitors). Other HIF pathway-targeting compounds may act through inhibition of HIFα/HIFβ dimerization, by inhibiting binding of HIF to the HRE of its target genes, or by inhibiting transcriptional activity of HIF (e.g., by inhibiting interaction of HIF with transcriptional coactivator p300) [13, 15, 19]. Despite this already significant amount of HIF pathway-targeting options, only a few of them have moved beyond the preclinical stage for therapeutic application in HCC [13, 15]. One of the most important limitations of these compounds is their lack of specificity towards a certain isoform of HIF, as the different isoforms, in addition to a substantial overlap, also have substantial differences in their array of target genes, and this may lead to opposing effects on the TME. Therefore, due to the relevance of the HIF pathway in several aspects of HCC development and progression, it is of great importance to further explore the therapeutic potential of isoform-specific HIF pathway-targeting strategies and their effect on the TME [13, 15, 19]. Here, we investigated the therapeutic potential of isoform-specific HIF-1α and HIF-2α antisense oligonucleotides (ASOs; provided by Ionis Pharmaceuticals), along with their effect on several TME-associated features, including inflammation and fibrosis, in a N,N-diethylnitrous amide (DEN)-induced HCC mouse model.

## RESULTS

### Pilot study for selection of optimal dosage regimen

**Figure 1:**
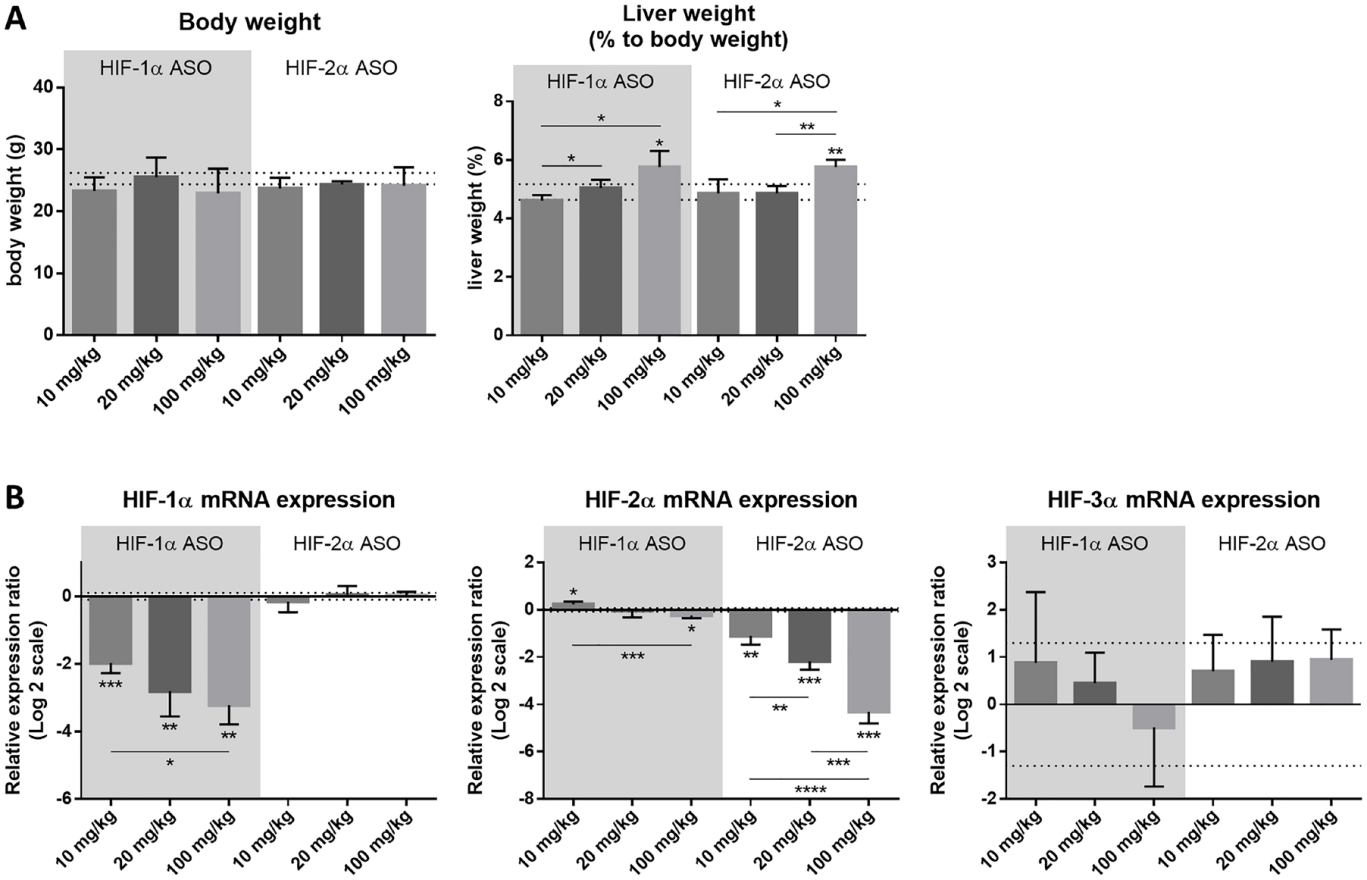
Efficacy and selectivity of different dosage regimens of HIF-1α and HIF-2α ASO. Mice were intraperitoneally injected with 10, 20 or 100 mg/kg HIF-1α or HIF-2α ASO, or 20 mg/kg scrambled ASO, twice per week for 2 weeks. (**A**) Body weight and relative liver weight (expressed as % to body weight) following treatment period. The upper and lower dashed lines represent mean ± SD of the scrambled ASO data. Mean ± SD of other data are represented as bars (*n* = 4 per treatment group). (**B**) Hepatic mRNA expression of the HIFα isoforms following treatment period. The upper and lower dashed lines represent mean ± SD of the log2-transformed 20 mg/kg scrambled ASO data. Log2-transformed mean ± SD of other data, relative to the log2-transformed mean of the 20 mg/kg scrambled ASO treatment group, are represented as bars (*n* = 4 per treatment group). ^*^ *p* < 0.05, ^**^ *p* < 0.01, ^***^ *p* < 0.001 and ^****^ *p* < 0.0001.

In order to define the optimal dosage regimen of the isoform-specific HIF-1α and HIF-2α ASOs used in further experiments, healthy mice were intraperitoneally injected with 10, 20 or 100 mg/kg HIF-1α or HIF-2α ASO, or 20 mg/kg scrambled ASO, twice per week for 2 weeks. Neither of the dosage regimens influenced the body weight of the mice. However, compared to scrambled ASO treatment, statistically significant hepatic enlargement was observed for both 100 mg/kg HIF-1α and HIF-2α ASO, while this was not observed for lower dosage regimens of both isoform-specific HIFα ASOs (Figure 1A).

Efficacy and selectivity of the HIFα ASOs was evaluated by comparing hepatic mRNA expression of all three HIFα isoforms in response to the different dosage regimens of HIF-1α and HIF-2α ASO, and scrambled control treatment. Both HIFα ASOs led to significant downregulation of mRNA expression of their respective target gene in a dose-dependent manner, compared to scrambled ASO treatment. The expression of HIF-1α was unaffected by HIF-2α ASO treatment, whereas HIF-1α ASO treatment showed a significant but neglectable effect (less than 2-fold change) on HIF-2α expression. Neither of the isoform-specific HIFα ASO treatments affected mRNA expression of the HIF-3α isoform (Figure 1B).

**Figure 2:**
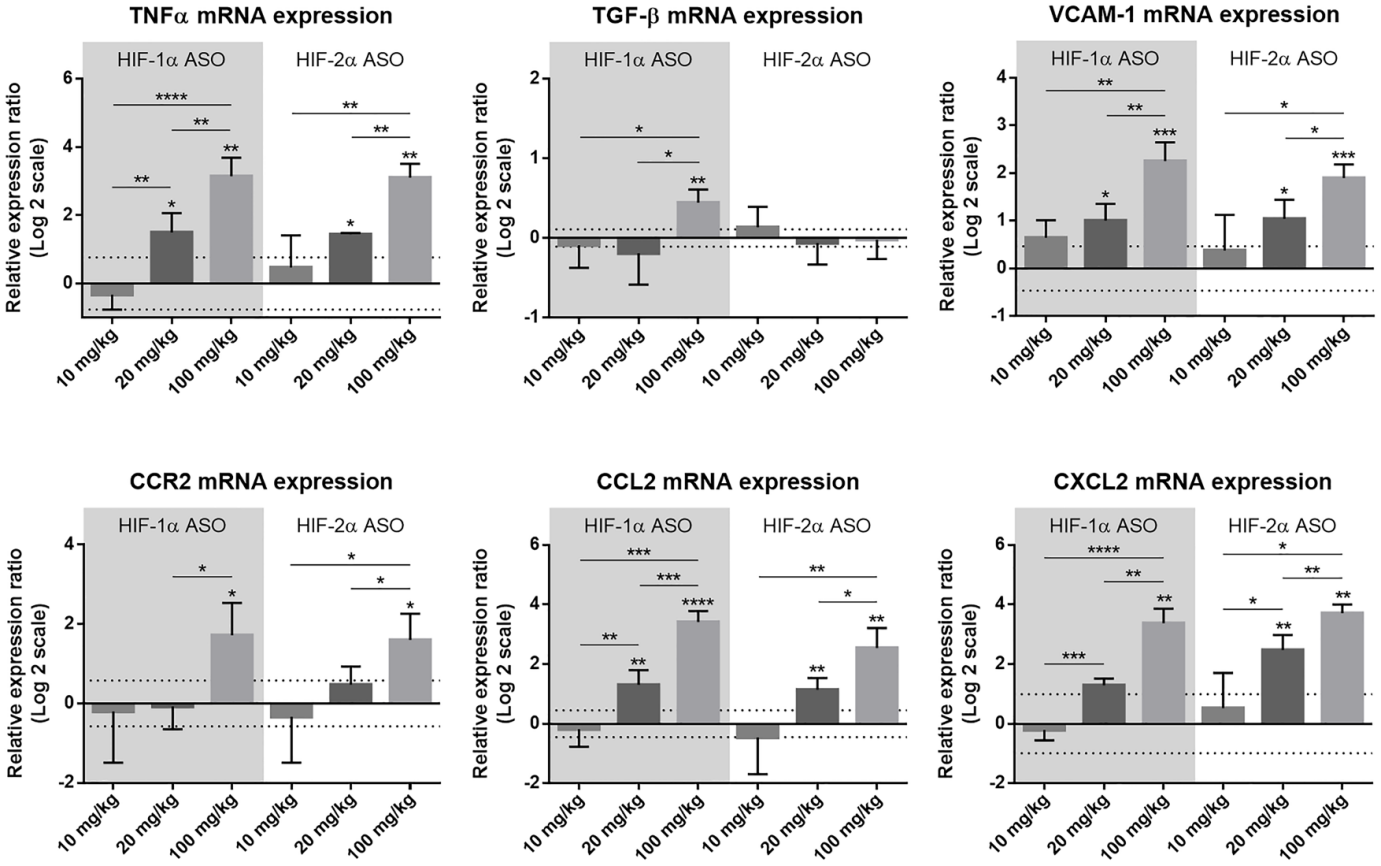
Effect of different dosage regimens of HIF-1α and HIF-2α ASO on hepatic expression of inflammatory markers. Mice were intraperitoneally injected with 10, 20 or 100 mg/kg HIF-1α or HIF-2α ASO, or 20 mg/kg scrambled ASO, twice per week for 2 weeks. Hepatic mRNA expression of the inflammatory markers TNFα, TGF-β, VCAM-1, CCR2, CCL2 and CXCL2 following treatment period is shown. The upper and lower dashed lines represent mean ± SD of the log2-transformed 20 mg/kg scrambled ASO data. Log2-transformed mean ± SD of other data, relative to the log2-transformed mean of the 20 mg/kg scrambled ASO treatment group, are represented as bars (n = 4 per treatment group). ^*^ *p* < 0.05, ^**^ *p* < 0.01, ^***^ *p* < 0.001 and ^****^ *p* < 0.0001.

Potential liver injury of the different dosage regimens was assessed via hepatic mRNA expression of several inflammation-associated markers. Compared to scrambled ASO treatment, expression of the pro-inflammatory cytokine tumor necrosis factor (TNF)α, vascular cell adhesion molecule (VCAM)-1 (mediates adhesion of several immune cells to vascular endothelium), C-C motif chemokine ligand (CCL)2 (mediates monocyte chemotaxis) and C-X-C motif chemokine ligand (CXCL)2 (involved in immune cell chemotaxis) was significantly upregulated following 20 mg/kg and 100 mg/kg HIF-1α and HIF-2α ASO treatment in a dose-dependent manner, whereas for the multifunctional cytokine TGF-β and C-C motif chemokine receptor (CCR)2 (mediates monocyte chemotaxis), hepatic expression was only increased following 100 mg/kg HIFα ASO treatment (Figure 2).

Based on the efficacy, as well as potential hepatotoxicity observed for the different treatment regimens, 20 mg/kg was selected as the optimal dose for further experiments.

### Selective and effective hepatic downregulation of target genes following HIFα ASO treatment in HCC mice

**Figure 3:**
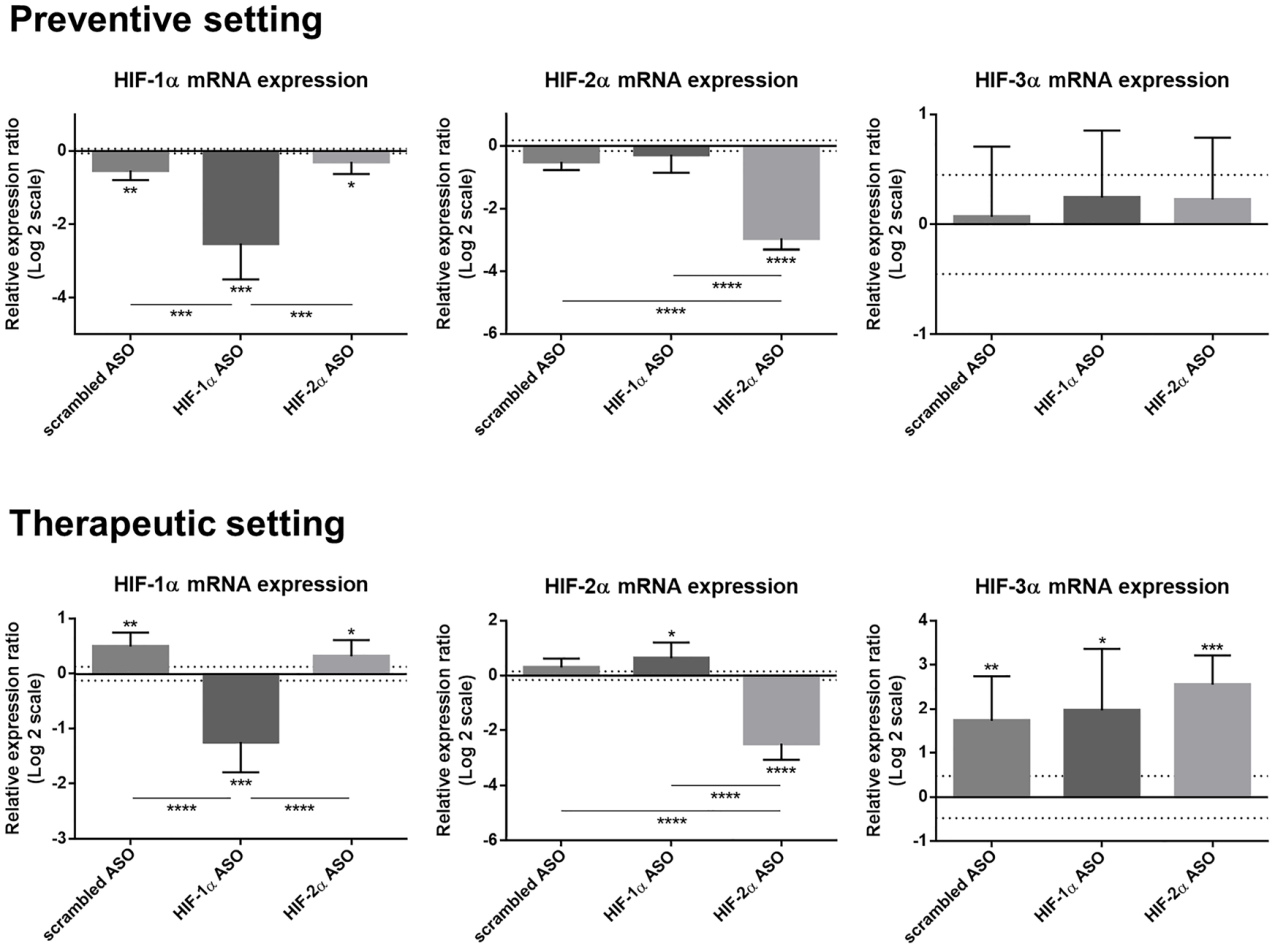
Efficacy and selectivity of preventive and therapeutic HIF-1α and HIF-2α ASO treatment in DEN-induced HCC mice. HCC was induced by weekly intraperitoneal DEN injection for 25 weeks. Control mice received weekly intraperitoneal 0.9% NaCl injection. DEN-treated mice were intraperitoneally injected with 20 mg/kg HIF-1α ASO, HIF-2α ASO or scrambled ASO twice per week, in either a preventive or a therapeutic setting. Control mice received scrambled ASO for the same duration of the experiment. Hepatic mRNA expression of the HIFα isoforms following preventive and therapeutic treatment is shown. The upper and lower dashed lines represent log2-transformed mean ± SD of the control mice. Bars represent log2-transformed mean ± SD of different treatment groups of DEN-treated mice, relative to the log2-transformed mean of the control mice (n = 7–9 per treatment group). ^*^ *p* < 0.05, ^**^ *p* < 0.01, ^***^ *p* < 0.001 and ^****^ *p* < 0.0001.

Efficacy and selectivity of isoform-specific HIFα ASO treatment was evaluated in the preventive and therapeutic setting in DEN-induced HCC mice. HIF-1α ASO effectively downregulated HIF-1α mRNA expression in both settings compared to healthy control mice and compared to other ASO treatment groups. HIF-1α expression was also slightly decreased after 15 weeks of scrambled and HIF-2α ASO administration, whereas therapeutic treatment of these ASOs resulted in minor upregulation of hepatic HIF-1α expression. HIF-2α ASO administration also selectively downregulated the expression of its target gene compared to all other groups. In addition, therapeutic HIF-1α ASO treatment resulted in minor upregulation of HIF-2α expression, compared to healthy control mice. In the therapeutic setting, all HCC mice showed increased expression of HIF-3α compared to healthy control mice without differences between treatment groups (Figure 3).

### Effect of HIFα ASO treatment on hepatocarcinogenesis

**Figure 4:**
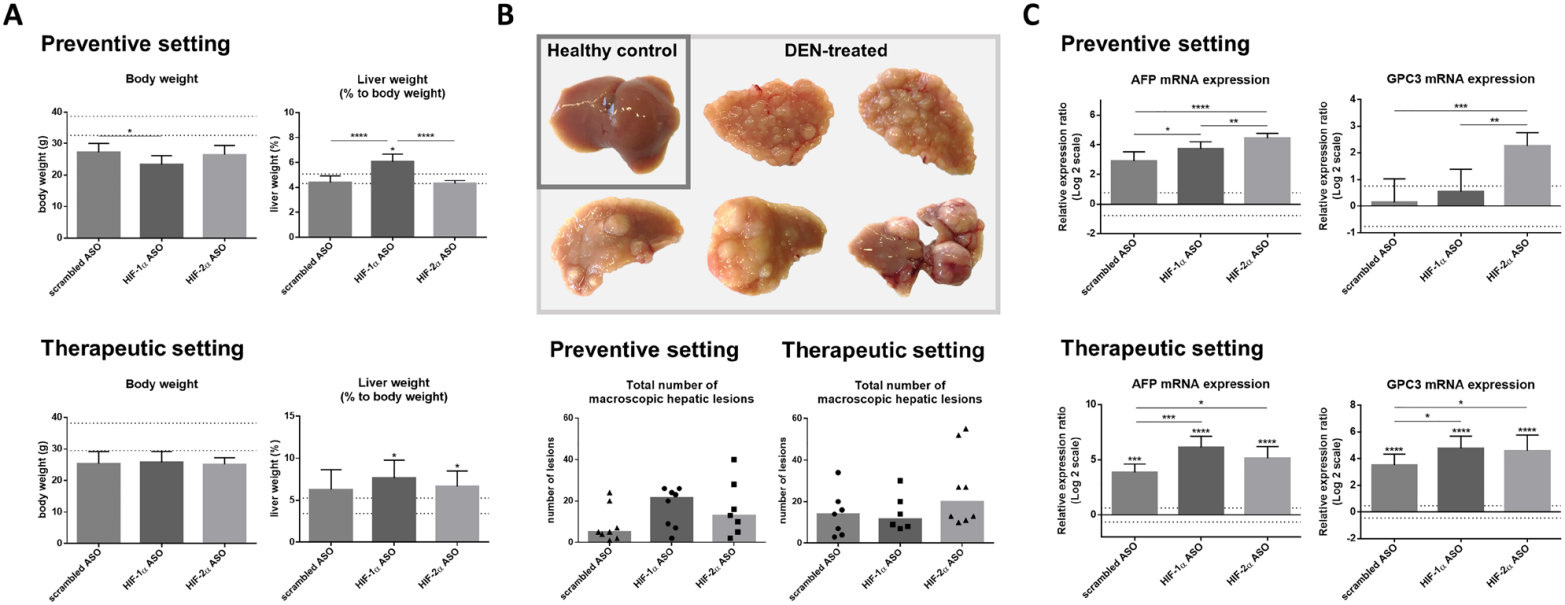
Effect of preventive and therapeutic HIF-1α and HIF-2α ASO treatment on hepatocarcinogenesis in DEN-induced HCC mice. HCC was induced by weekly intraperitoneal DEN injection for 25 weeks. Control mice received weekly intraperitoneal 0.9% NaCl injection. DEN-treated mice were intraperitoneally injected with 20 mg/kg HIF-1α ASO, HIF-2α ASO or scrambled ASO twice per week, in either a preventive or a therapeutic setting. Control mice received scrambled ASO for the same duration of the experiment. (**A**) Body weight and relative liver weight (expressed as % to body weight) following preventive and therapeutic treatment. The upper and lower dashed lines represent mean ± SD of the control mice. Bars represent mean ± SD of different treatment groups of DEN-treated mice (*n* = 6–8 per treatment group). (**B**) Upper part: Hepatic tissue of healthy control mice and DEN-treated mice. Lower part: Total number of macroscopic hepatic lesions following preventive and therapeutic treatment. Data are represented as individual values with the median (n = 6–8 per treatment group). (**C**) Hepatic mRNA expression of the HCC tumor markers AFP and GPC3 following preventive and therapeutic treatment. The upper and lower dashed lines represent log2-transformed mean ± SD of the control mice. Bars represent log2-transformed mean ± SD of different treatment groups of DEN-treated mice, relative to the log2-transformed mean of the control mice (n = 7–9 per treatment group). ^*^ *p* < 0.05, ^**^ *p* < 0.01, ^***^ *p* < 0.001 and ^****^ *p* < 0.0001.

The effect of isoform-specific HIFα ASOs on the development of HCC (preventive setting) and their potential as therapeutic strategy for HCC (therapeutic setting) were assessed in the established DEN-induced HCC mouse model, which is known to be characterized by hypoxia and induction of the HIF pathway [21–22]. Regardless of the ASO treatment, all DEN-injected mice showed lower body weight at the end of the experiment compared to healthy control mice, however without significance. Only preventive HIF-1α ASO administration resulted in significant hepatic enlargement in HCC mice compared to healthy control mice and other treatment groups, whereas in the therapeutic setting, both HIF-1α and HIF-2α ASO treatment resulted in significant hepatomegaly (Figure 4A).

To investigate the effect of isoform-specific HIF-1α and HIF-2α ASO on HCC tumorigenesis, the number of macroscopically visible hepatic tumoral lesions in the different treatment groups was evaluated. DEN treatment led to a rough nodular hepatic surface with multiple macroscopic lesions in all treatment groups. However, no significant effect of HIFα ASO treatment could be observed. The overall number of tumoral lesions was higher in HCC mice euthanized 28 weeks following first DEN injection (therapeutic setting), compared to mice that were euthanized three weeks earlier (preventive setting) (Figure 4B).

The effect of isoform-specific HIFα ASO treatment on HCC tumorigenesis was further investigated via assessment of hepatic mRNA expression of the HCC tumor markers α-fetoprotein (AFP) and glypican-3 (GPC3). None of the HCC mice that were euthanized 25 weeks following first DEN injection showed significantly increased expression of HCC markers compared to healthy control mice, whereas all mice euthanized 3 weeks later did. With the exception of GPC3 in 15 weeks HIF-1α ASO treated mice, HCC markers were upregulated in HIFα ASO-treated HCC mice compared to scrambled ASO-treated HCC mice, both in the preventive and therapeutic setting (Figure 4C).

### HIF-1α ASO treatment induces a pro-inflammatory TME in DEN-induced HCC mice

**Figure 5:**
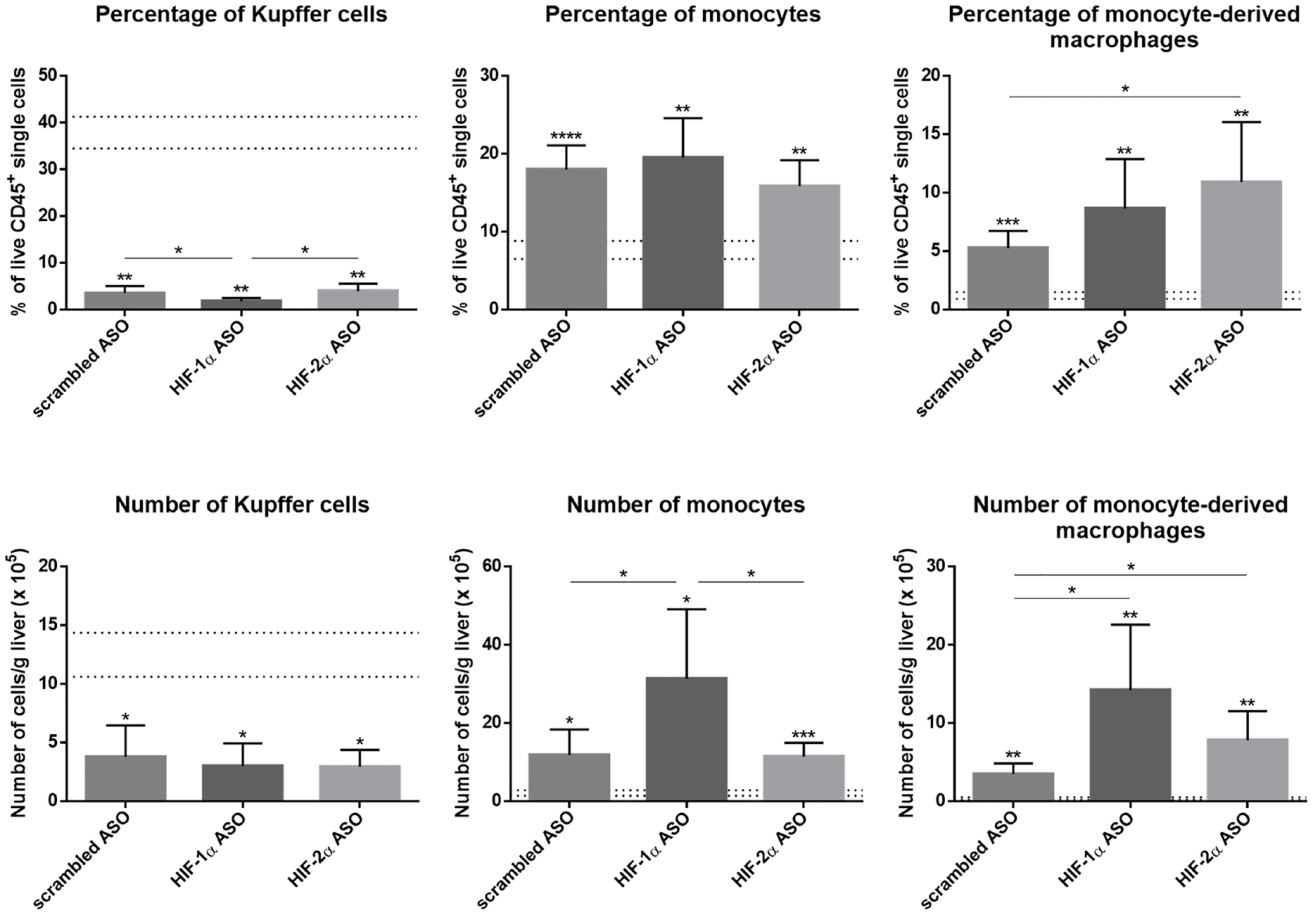
Effect of therapeutic HIF-1α and HIF-2α ASO treatment on hepatic macrophage pool in DEN-induced HCC mice. HCC was induced by weekly intraperitoneal DEN injection for 25 weeks. Control mice received weekly intraperitoneal 0.9% NaCl injection. DEN-treated mice were intraperitoneally injected with 20 mg/kg HIF-1α ASO, HIF-2α ASO or scrambled ASO twice per week, in a therapeutic setting. Control mice received scrambled ASO for the same duration of the experiment. Upper part: Percentage of CD11b^+^Ly6C^-^F4/80^+^Tim4^+^ Kupffer cells, CD11b^+^Ly6C^+^F4/80^+^Tim4^-^ monocytes and CD11b^+^Ly6C^-^F4/80^+^Tim4^-^ monocyte-derived macrophages in live CD45^+^ single cell gate following treatment, measured by flow cytometry. The upper and lower dashed lines represent mean ± SD of the control mice. Bars represent mean ± SD of different treatment groups of DEN-treated mice (n = 6–7 per treatment group). ^*^ *p* < 0.05, ^**^ *p* < 0.01, ^***^ *p* < 0.001 and ^****^ *p* < 0.0001. Lower part: Number of live CD45^+^CD11b^+^Ly6C^-^F4/80^+^Tim4^+^ Kupffer cells, CD45^+^CD11b^+^Ly6C^+^F4/80^+^Tim4^-^ monocytes and CD45^+^CD11b^+^Ly6C^-^F4/80^+^Tim4^-^ monocyte-derived macrophages per gram liver tissue following treatment, measured by flow cytometry. The upper and lower dashed lines represent mean ± SD of the control mice. Bars represent mean ± SD of different treatment groups of DEN-treated mice (n = 6–7 per treatment group). ^*^ *p* < 0.05, ^**^ *p* < 0.01, ^***^ *p* < 0.001 and ^****^ *p* < 0.0001.

The effect of HIF-1α and HIF-2α ASO treatment on the inflammatory component of the TME in the DEN-induced HCC mouse model was first assessed via flow cytometric analysis of the hepatic macrophage pool. As previously published by our group, the percentage and number of CD11b+Ly6C-F4/80+Tim4+ Kupffer cells (KCs) was significantly decreased in HCC mice, independent from treatment [23]. As expected, DEN-induced HCC resulted in a marked increase of CD11b+Ly6C+Ly6G- monocytes and CD11b+Ly6C-F4/80+Tim4- monocyte-derived macrophages (MoMfs) in the liver, which was most pronounced in mice treated with HIF-1α ASO (Figure 5).

**Figure 6:**
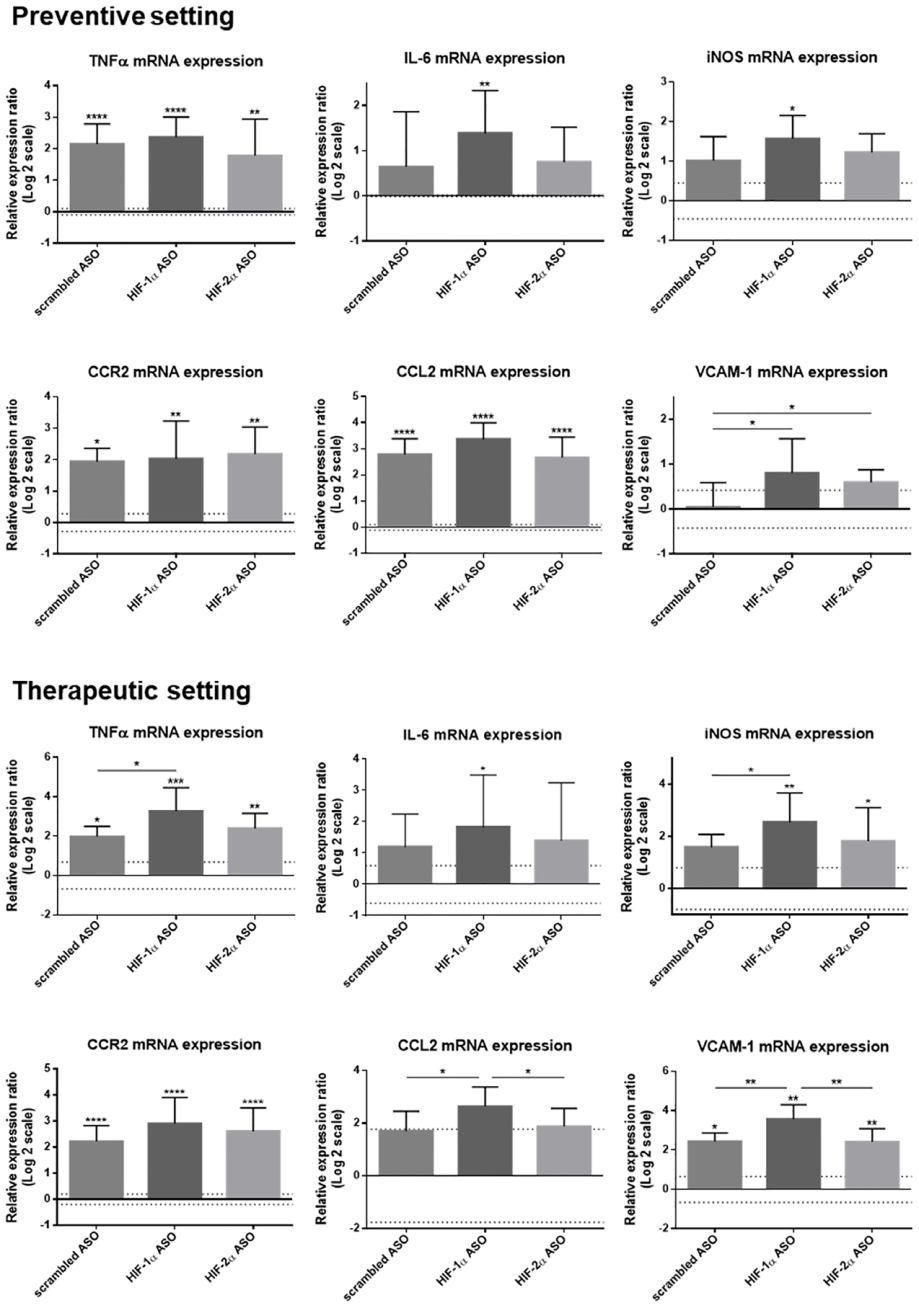
Effect of preventive and therapeutic HIF-1α and HIF-2α ASO treatment on hepatic mRNA expression of inflammatory markers in DEN-induced HCC mice. HCC was induced by weekly intraperitoneal DEN injection for 25 weeks. Control mice received weekly intraperitoneal 0.9% NaCl injection. DEN-treated mice were intraperitoneally injected with 20 mg/kg HIF-1α ASO, HIF-2α ASO or scrambled ASO twice per week, in either a preventive or a therapeutic setting. Control mice received scrambled ASO for the same duration of the experiment. Hepatic mRNA expression of several inflammatory markers following preventive and therapeutic treatment is shown. The upper and lower dashed lines represent log2-transformed mean ± SD of the control mice. Bars represent log2-transformed mean ± SD of different treatment groups of DEN-treated mice, relative to the log2-transformed mean of the control mice (n = 7–9 per treatment group). ^*^ *p* < 0.05, ^**^ *p* < 0.01, ^***^ *p* < 0.001 and ^****^ *p* < 0.0001.

The effect of isoform-specific HIFα ASO treatment on inflammation was further assessed via hepatic expression of several inflammatory markers, both at mRNA and protein level. DEN-induced HCC resulted in significant upregulation of the mRNA levels of the pro-inflammatory markers TNFα, CCR2, CCL2 and VCAM-1 (the latter two only in respectively the preventive and therapeutic group), and, in most settings, this was most pronounced in mice treated with HIF-1α ASO. In addition, only HIF-1α ASO-treated mice showed significantly increased expression of the pro-inflammatory cytokine interleukin (IL)-6 and inducible nitric oxide synthase (iNOS) (Figure 6).

**Figure 7:**
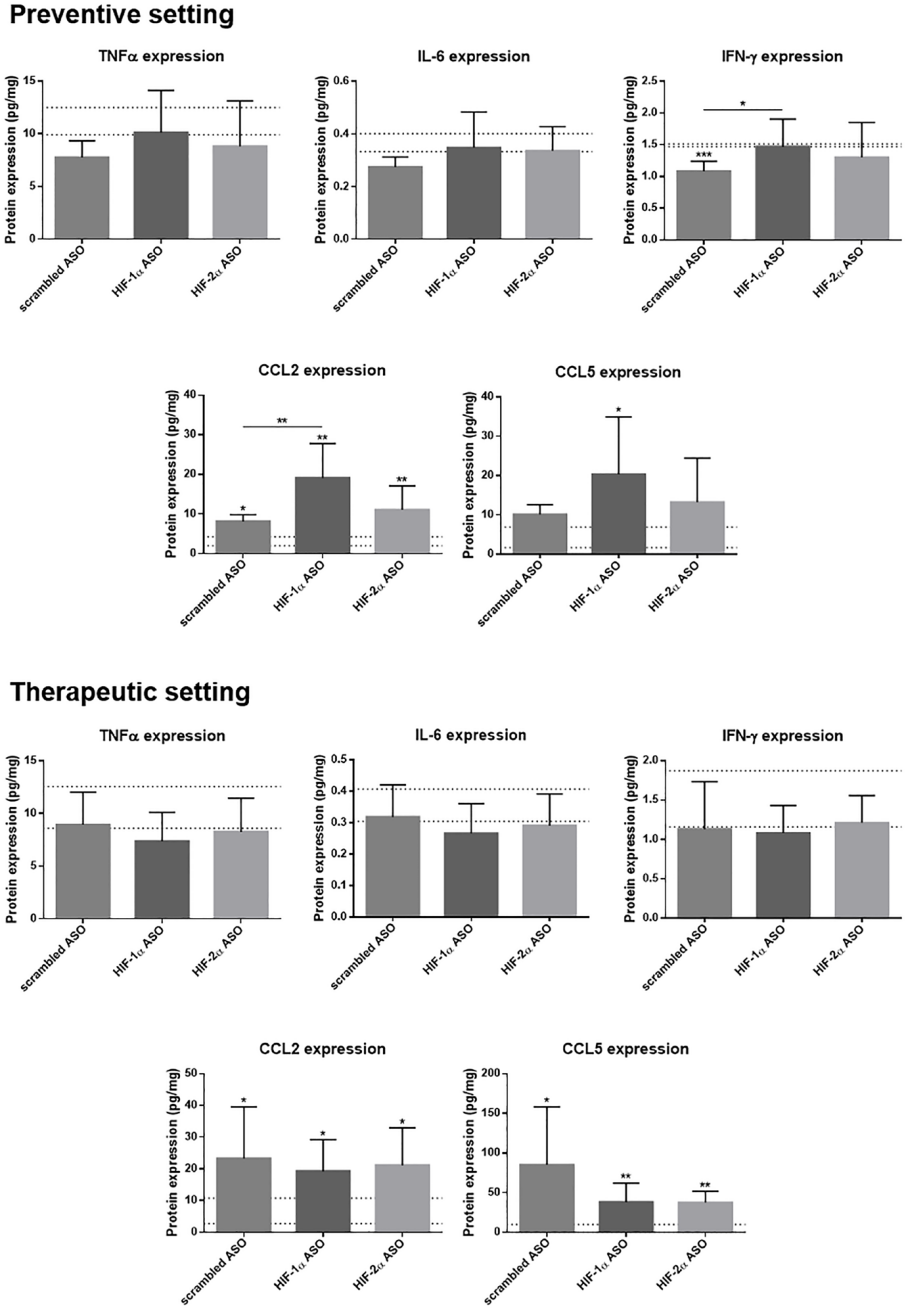
Effect of preventive and therapeutic HIF-1α and HIF-2α ASO treatment on hepatic protein expression of inflammatory markers in DEN-induced HCC mice. HCC was induced by weekly intraperitoneal DEN injection for 25 weeks. Control mice received weekly intraperitoneal 0.9% NaCl injection. DEN-treated mice were intraperitoneally injected with 20 mg/kg HIF-1α ASO, HIF-2α ASO or scrambled ASO twice per week, in either a preventive or a therapeutic setting. Control mice received scrambled ASO for the same duration of the experiment. Hepatic protein expression of several inflammatory markers following preventive and therapeutic treatment is shown. The upper and lower dashed lines represent mean ± SD of the control mice. Bars represent mean ± SD of different treatment groups of DEN-treated mice (n = 7–9 per treatment group). ^*^ *p* < 0.05, ^**^ *p* < 0.01, ^***^ *p* < 0.001 and ^****^ *p* < 0.0001.

While we did not observe increased protein levels of TNFα, IL-6 and interferon (IFN)-γ, nor effect of ASO treatment on these protein levels, in mice with HCC, CCL2 and CCL5 protein levels, responsible for leukocyte recruitment, were significantly increased in HCC mice and this was most pronounced for HIF-1α ASO-treated mice in the preventive setting (Figure 7).

### HIFα ASO treatment aggravates fibrosis in DEN-induced HCC mice

**Figure 8:**
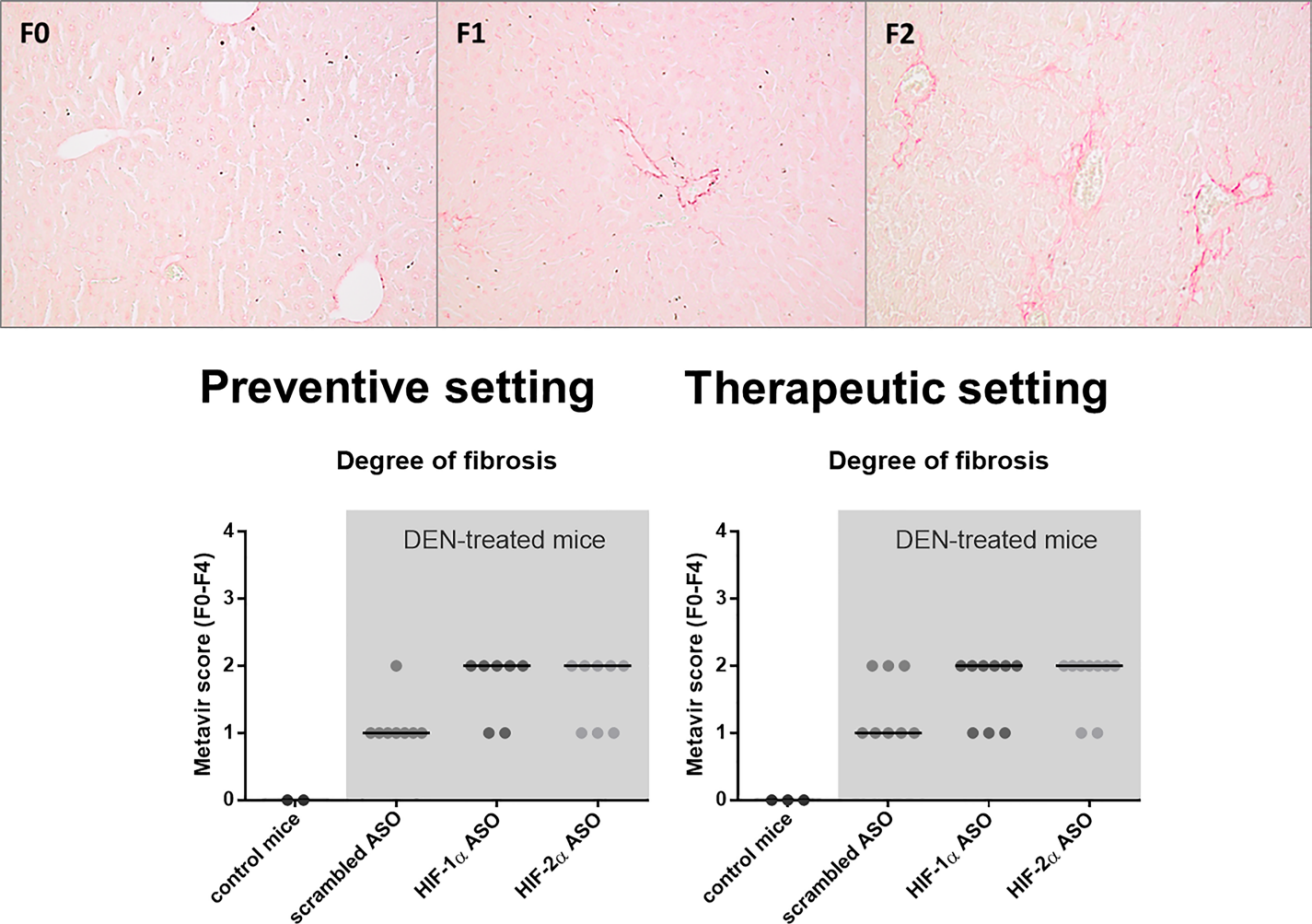
Effect of preventive and therapeutic HIF-1α and HIF-2α ASO treatment on liver fibrosis in DEN-induced HCC mice. HCC was induced by weekly intraperitoneal DEN injection for 25 weeks. Control mice received weekly intraperitoneal 0.9% NaCl injection. DEN-treated mice were intraperitoneally injected with 20 mg/kg HIF-1α ASO, HIF-2α ASO or scrambled ASO twice per week, in either a preventive or a therapeutic setting. Control mice received scrambled ASO for the same duration of the experiment. Upper part: Representative histological images of Sirius Red-stained liver sections with Metavir fibrosis stage F0 (no fibrosis), F1 (portal fibrosis without septa) and F2 (portal fibrosis with few septa). Magnification 200×. Lower part: Degree of hepatic fibrosis following preventive and therapeutic treatment, assessed through histological analysis of Sirius Red-stained liver sections using the Metavir scoring system. Data are represented as individual values with the median (n = 7–9 per HCC treatment group).

In the vast majority of the cases, HCC develops on a fibrotic background. As hypoxia, and more specifically the HIF pathway, plays an important role in HCC-associated fibrogenesis, we investigated the effect of isoform-specific HIF-1α and HIF-2α ASO treatment on fibrosis in the DEN-induced HCC mouse model [24, 25]. DEN-induced hepatocarcinogenesis only resulted in minor fibrosis, with Metavir fibrosis stages ranging from F1 to F2, as previously published [26]. Remarkably, HIFα ASO treatment aggravated fibrosis in the livers of DEN-treated mice, both in the preventive and therapeutic setting (Figure 8).

**Figure 9:**
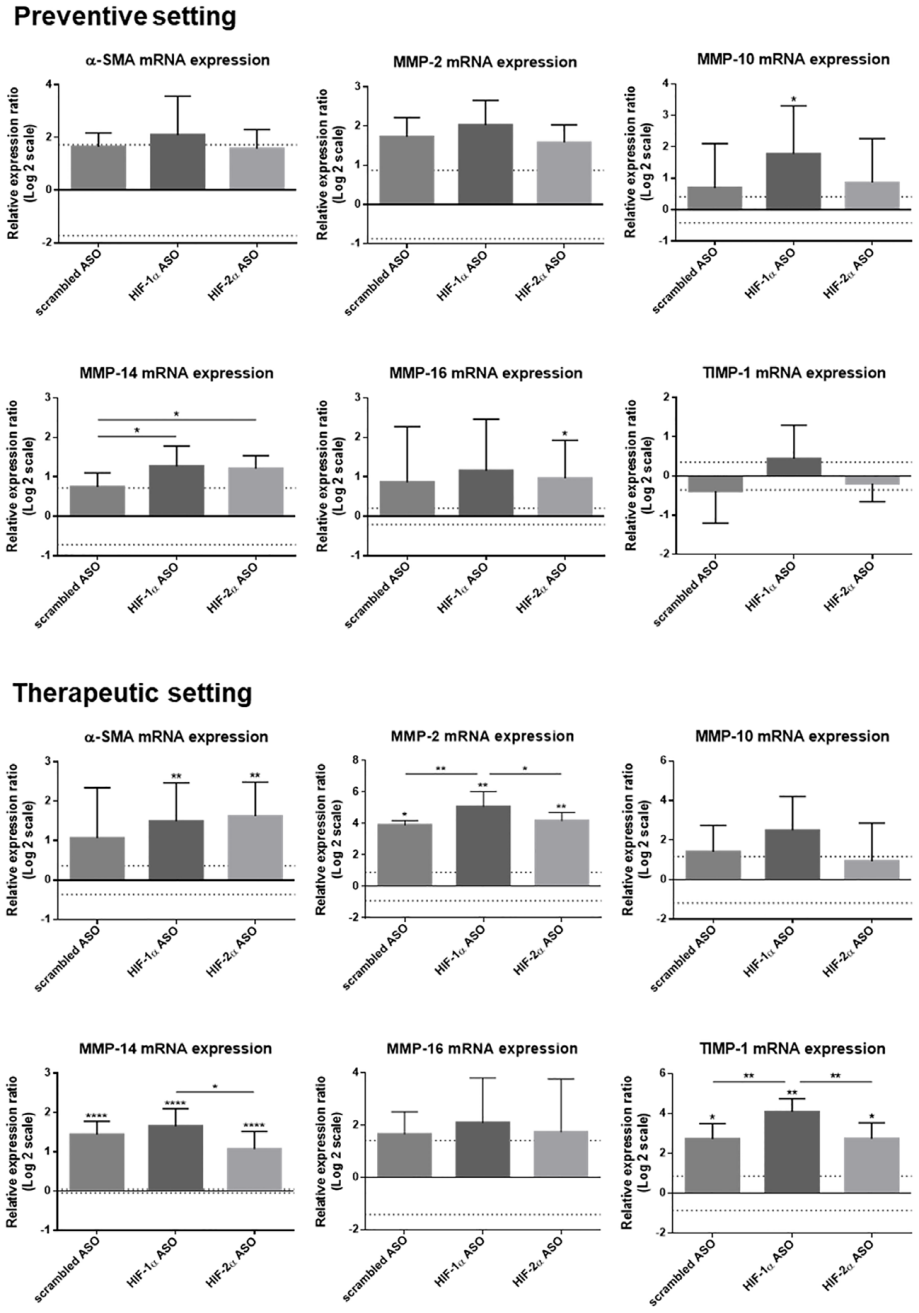
Effect of preventive and therapeutic HIF-1α and HIF-2α ASO treatment on fibrotic markers in the liver of DEN-induced HCC mice. HCC was induced by weekly intraperitoneal DEN injection for 25 weeks. Control mice received weekly intraperitoneal 0.9% NaCl injection. DEN-treated mice were intraperitoneally injected with 20 mg/kg HIF-1α ASO, HIF-2α ASO or scrambled ASO twice per week, in either a preventive or a therapeutic setting. Control mice received scrambled ASO for the same duration of the experiment. Hepatic mRNA expression of fibrotic markers following preventive and therapeutic treatment is shown. The upper and lower dashed lines represent log2-transformed mean ± SD of the control mice. Bars represent log2-transformed mean ± SD of different treatment groups of DEN-treated mice, relative to the log2-transformed mean of the control mice (*n* = 7–9 per treatment group). ^*^ *p* < 0.05, ^**^ *p* < 0.01, ^***^ *p* < 0.001 and ^****^ *p* < 0.0001.

The effect of isoform-specific HIFα ASO treatment on liver fibrosis was further assessed via hepatic mRNA expression of several fibrotic markers. The expression of α-smooth muscle actin (α-SMA), which is a marker for the formation of extracellular matrix (ECM)-producing myofibroblasts, confirmed our histological analysis with significant upregulation in HIFα ASO-treated HCC mice. Furthermore, several matrix metalloproteinases (MMPs) and tissue inhibitors of metalloproteinases (TIMPs) involved in fibrogenesis and HCC progression were upregulated in the livers of HCC mice, which was again most pronounced following HIFα ASO treatment (Figure 9).

## DISCUSSION

Due to the worldwide increase of the incidence of some of its major etiological risk factors, including chronic alcohol abuse and NAFLD, HCC is a growing health problem [1, 27–28]. It is often only diagnosed at an advanced stage, at which current treatment options are limited and unsatisfactory [3, 5, 29]. Therefore, promising novel therapeutic targets need to be explored [7]. As the HIF pathway regulates multiple cancer hallmarks and consequently has a key role in shaping the HCC TME, targeting this hypoxic response pathway represents an attractive strategy for HCC treatment. To date, only a small portion of HIF pathway-targeting compounds has moved beyond the preclinical stage. A common and important limitation is the lack of specificity towards a certain HIF isoform [13, 15]. Therefore, this study aimed to explore the therapeutic potential of isoform-specific HIF pathway targeting by means of selective HIF-1α and HIF-2α ASOs. In addition to their effect on HCC development and progression, the influence on the inflammatory and fibrotic component of the TME was assessed. In our study, HIFα ASO treatment selectively downregulated its target gene, but did not exert a beneficial effect on hepatocarcinogenesis, induced a pro-inflammatory TME and aggravated fibrosis in the liver of DEN-induced HCC mice.

Three HIFα isoforms (HIF-1α, HIF-2α and HIF-3α) have been described so far [30]. Increased expression of both HIF-1α and HIF-2α has been observed in several chronic liver diseases, including alcoholic liver disease (ALD), NAFLD and HCC [31]. Especially HIF-1α expression has been shown to be positively correlated with poor prognosis, tumor grade, metastasis and lower overall survival rate [15, 31–32]. The observed upregulation of HIF-1α in HCC mice euthanized 28 weeks following first DEN injection (therapeutic setting), compared to mice that were euthanized three weeks earlier (preventive setting), along with the increased number of macroscopic tumoral lesions, indeed indicates that HIF-1α expression increases in advanced HCC. The contribution of HIF-2α in HCC pathogenesis is less clear, as both tumor promoting and tumor suppressing results have been published [31, 32]. Observed discrepancies in the involvement of HIF-1α and HIF-2α in HCC development and progression can be explained by the fact that, in addition to a substantial overlap of target genes, they each also regulate a distinct set of genes [30, 32].

A dose-finding study was set up in order to define the optimal dosage regimen of the isoform-specific HIFα ASOs. Both HIFα ASOs selectively downregulate mRNA expression of their respective target gene. Only for HIF-2α ASO treatment, dose increase above 20 mg/kg resulted in more effective HIF-2α mRNA downregulation. Furthermore, for both HIFα ASOs, the 100 mg/kg dosage regimen resulted in significant hepatomegaly and upregulation of the mRNA expression of several inflammation-associated markers, including TNFα, VCAM-1, CCR2, CCL2 and CXCL2, compared to lower dosage regimens, indicating dose-dependent hepatotoxicity. Consequently, based on its efficacy and safety, we opted for a dosage regimen of 20 mg/kg twice per week, for both HIF-1α and HIF-2α ASO, in subsequent experiments.

In order to investigate the effect of isoform-specific HIFα inhibition on HCC tumorigenesis, a DEN-induced HCC mouse model was employed. This experimental HCC mouse model was selected due to its physiologically relevant TME and immune modifications related to HCC development and progression, as long-term repetitive DEN administration induces both chronic liver inflammation and mild fibrosis [33]. DEN injections resulted in formation of macroscopic hepatic lesions in 100% of the cases. Tumor formation was associated with upregulated mRNA expression of the established HCC tumor markers AFP and GPC3 [34–35]. Flow cytometric analysis of the hepatic macrophage pool confirmed KC depletion, simultaneous Ly6C^+^ monocyte infiltration and increased MoMfs [23, 36]. Hepatic infiltration of immune cells and establishment of a pro-inflammatory TME was further confirmed via upregulated expression of several inflammatory markers, including TNFα, CCR2, CCL2, CCL5 and VCAM-1, at either mRNA or protein level [37–40]. Portal fibrosis without septa (Metavir fibrosis stage F1) was observed in the peritumoral hepatic tissue, in both treatment settings. Furthermore, the observed upregulation of the hepatic mRNA expression of MMP-2, MMP-14 and TIMP-1 is in line with the establishment of a fibrotic TME following chronic DEN administration [41–43]. Thus, as the DEN-induced HCC mouse model mimics the human HCC-associated TME in several aspects, it is a suitable experimental HCC model for investigation of the therapeutic potential of our isoform-specific HIFα ASOs.

As macroscopic HCC lesions are only observed following approximately 20 weeks of weekly 35 mg/kg DEN injections, initiation of HIFα ASO treatment 10 weeks following the first DEN injection enables investigation of its effect on HCC initiation and development (preventive setting) [26, 44]. Starting HIFα ASO treatment 20 weeks following first DEN administration allows to assess its effect on the progression of HCC (therapeutic setting). As it was the case in the dose-finding study, in both treatment settings, isoform-specific HIF-1α and HIF-2α ASO effectively and selectively downregulated hepatic mRNA expression of their target gene. Biometric data however gave some indications of worse general and hepatic condition of the HIFα ASO-treated mice, as in some treatment groups, relative to the scrambled ASO treatment group, additional weight loss and/or hepatomegaly was observed.

Regarding the effect of specific HIF-1α and HIF-2α inhibition on HCC tumorigenesis, preventive treatment did not inhibit or slow down tumor development in our HCC mouse model, as macroscopic lesions were present in 100% of the ASO-treated mice and furthermore, were more numerous compared to the scrambled ASO treatment group. Moreover, following both preventive and therapeutic HIF-1α and HIF-2α ASO treatment, upregulated hepatic mRNA expression of AFP and/or GPC3 was observed, as compared to scrambled ASO treatment. This finding is supported by previous research which showed that HIF-1α overexpression downregulates transcriptional activity of the AFP and GPC3 genes in HCC cells, through competition with the oncogenic transcription factor c-Myc [45–46]. Thus, in this case, experimental HIF-1α inhibition might result in c-Myc-dependent upregulation of AFP and GPC3 mRNA expression. In addition, HIF-2α-mediated inhibition of tumor growth in HCC has also been previously described [47]. Consequently, the observed absence of beneficial effect of isoform-specific HIFα inhibition on the development of macroscopic HCC lesions, together with upregulated hepatic mRNA expression of HCC tumor markers, discourage the use of HIF-1α and HIF-2α as targets for the treatment of HCC.

Despite the negative effects of isoform-specific HIFα ASO treatment on HCC tumorigenesis in the DEN-induced HCC mouse model, it is of great importance to create a better understanding of the distinct influences the different HIFα isoforms exert on the HCC-associated TME. As chronic inflammation and fibrosis are two major features of human HCC, and are shown to play an essential role in its development and progression, we also investigated the effect of isoform-specific HIFα inhibition on both the inflammatory and fibrotic component of the TME associated with experimental DEN-induced HCC [48, 49].

Both HIF-1α and HIF-2α have been demonstrated to play essential roles in monocyte recruitment and macrophage differentiation in many cancers, including HCC [37, 50–52]. However, in our HCC model, isoform-specific HIFα inhibition resulted in increased Ly6C^+^ monocyte infiltration and/or MoMf differentiation. The pro-inflammatory effect of HIFα inhibition on the HCC TME was further demonstrated by upregulated hepatic mRNA and/or protein expression of several inflammation-related markers, namely the pro-inflammatory cytokines TNFα, IL-6 and IFN-γ, the M1-like macrophage marker iNOS, and the immune cell infiltration-related markers CCL2, CCL5 and VCAM-1.

Both HIF-1α and HIF-2α transcriptional activity have also been demonstrated to directly or indirectly contribute to fibrogenesis [24, 31, 53, 54]. However, in both treatment settings, isoform-specific HIFα inhibition resulted in an increased extent of fibrosis in the peritumoral hepatic tissue. This pro-fibrotic effect of HIFα inhibition on the HCC TME was further demonstrated by upregulated hepatic mRNA expression of several fibrotic markers, namely α-SMA, MMP-2, MMP-10, MMP-14, MMP-16 and/or TIMP-1.

The observed negative effects of both isoform-specific HIF-1α and HIF-2α ASO treatment on HCC tumorigenesis in our DEN-induced HCC mouse model, discourage the use of HIF-1α and HIF-2α as targets for the treatment of HCC. Furthermore, the pro-inflammatory and pro-fibrotic effects on the HCC TME also raise questions about the use of HIFα ASOs, in the dosing regimen we have investigated, in other cancers, with respect to potential hepatotoxicity. Further preclinical studies testing dosing strategies might indicate if lower doses of isoform-specific HIFα ASOs can be used without potential harm and might be of interest as anti-cancer therapy.

## MATERIALS AND METHODS

### Animals

Male wild type 129/Sv mice were purchased from Janvier Labs (Le Genest-Saint-Isle, France) at 4 weeks of age and housed in open cages in a temperature-controlled room at 20°C with a 12 hour light/dark cycle at the laboratory animal facility of the Faculty of Medicine and Health Sciences (Ghent University, Ghent, Belgium). Mice were given *ad libitum* access to food (mouse maintenance chow; Carfil Quality – Labofood, Oud-Turnhout, Belgium) and water. Mice were acclimatized under controlled conditions for one week prior to the experiments. All mice received care in accordance with the “Guide for the Care and Use of Laboratory Animals” and the Belgian national guidelines for animal protection. HCC was induced at the age of 5 weeks via weekly intraperitoneal injections of 35 mg/kg DEN (Sigma-Aldrich, Machelen, Belgium), dissolved in 0.9% sodium chloride (NaCl; B. Braun, Machelen, Belgium) for 25 weeks. Control mice received weekly intraperitoneal injections of 0.9% NaCl. Mice were monitored for weight loss and other external signs of disease or discomfort. This study was approved by the Ethical Committee of Experimental Animals at the Faculty of Medicine and Health Sciences of Ghent University (ECD 18/125).

### Dose-finding study

In order to define the optimal dosage regimen of the isoform-specific ASOs used in our preventive and therapeutic studies, 8-week-old male wild type 129/Sv mice were intraperitoneally injected twice per week for 2 weeks with 10, 20 or 100 mg/kg HIF-1α ASO, 10, 20 or 100 mg/kg HIF-2α ASO, or 20 mg/kg scrambled ASO (all provided by Ionis Pharmaceuticals, Carlsbad, California, USA), dissolved in phosphate-buffered saline (PBS; Gibco, Thermo Fisher Scientific, Merelbeke, Belgium). Afterwards, mice were sacrificed, the liver was isolated (see "Tissue sampling") and expression of the different HIFα isoforms and several inflammatory markers was analyzed via quantitative reverse transcription-polymerase chain reaction (RT-qPCR).

### Preventive and therapeutic treatment

**Figure 10:**
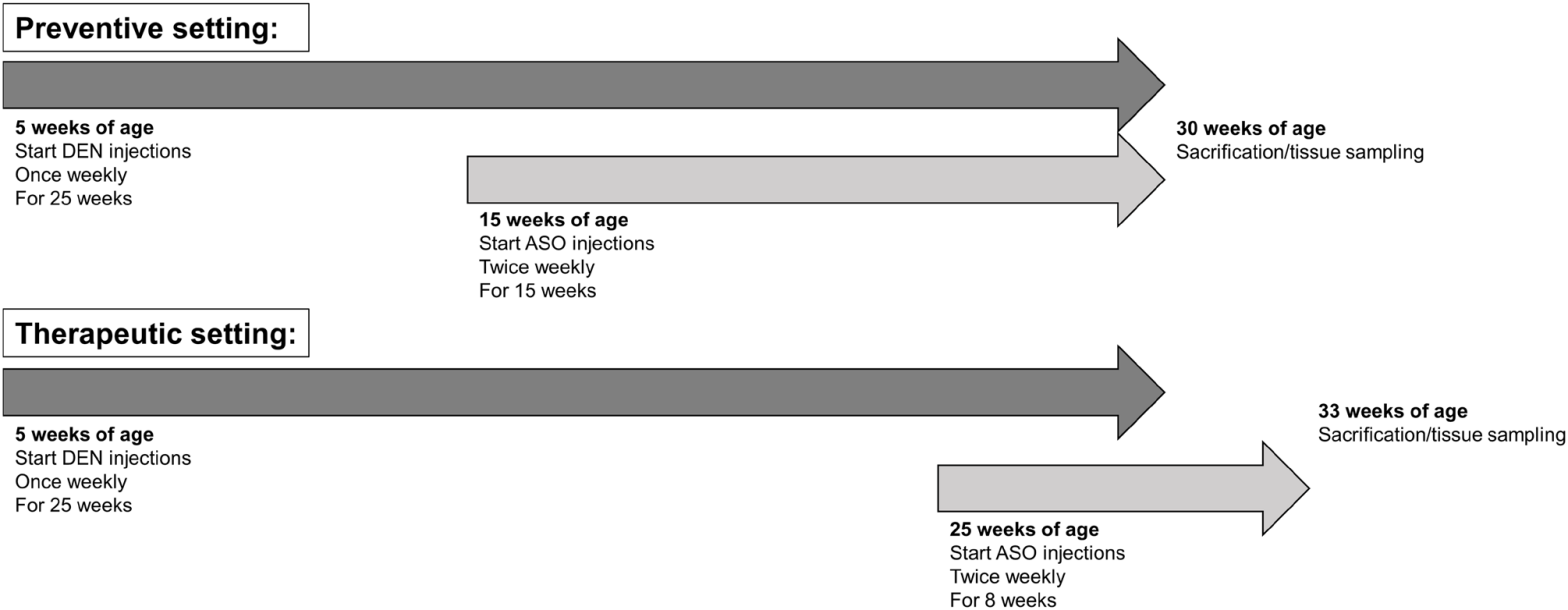
Preventive and therapeutic treatment of ASOs in experimental HCC. HCC was induced by weekly intraperitoneal DEN injection for 25 weeks. Control mice received weekly intraperitoneal 0.9% NaCl injection. DEN-treated mice were intraperitoneally injected with 20 mg/kg HIF-1α ASO, HIF-2α ASO or scrambled ASO twice per week. In the preventive setting, ASO injections started 10 weeks following the first DEN injection (at 15 weeks of age) and persisted for a period of 15 weeks. In the therapeutic setting, ASO injections started 20 weeks following the first DEN injection (at 25 weeks of age) and persisted for a period of 8 weeks. Control mice received scrambled ASO for the same duration of the experiment. Following respective treatment periods, mice were sacrificed and the liver was isolated for further analyses.

DEN-treated mice were intraperitoneally injected twice per week with 20 mg/kg HIF-1α ASO, HIF-2α ASO or scrambled ASO, dissolved in PBS, in either a preventive or therapeutic setting. In the preventive setting, ASO injections started 10 weeks after the first DEN injection (at 15 weeks of age) and persisted for a period of 15 weeks. In the therapeutic setting, ASO injections started 20 weeks after the first DEN injection (at 25 weeks of age) and persisted 8 weeks. These treatment regimens are based on previous publications reporting HCC development by weekly DEN injections [26, 44]. Control mice received intraperitoneal injections of scrambled ASO twice per week. Following treatment, mice were sacrificed and the liver was divided for further analyses (Figure 10).

### Tissue sampling

Mice were anesthetized via intraperitoneal injection of ketamine (60 mg/kg; Dechra Veterinary Products, Lille, Belgium) and xylazine (6 mg/kg; Kela, Sint-Niklaas, Belgium), and euthanized by cervical dislocation. Two pieces of the liver were isolated; one was fixed in 4% phosphate-buffered formaldehyde solution (Klinipath, Olen, Belgium) for histological analysis, and one was incubated in RNAlater (Ambion, Thermo Fisher Scientific), snap frozen in liquid nitrogen and stored at –80°C until further processing for RT-qPCR. In mice belonging to the therapeutic treatment groups, the remaining liver was perfused with PBS, isolated, weighed, chopped into small pieces, further dissociated using gentleMACS Dissociator (Miltenyi Biotec, Leiden, The Netherlands), and incubated for 20 minutes in 1 mg/mL Collagenase A (Sigma-Aldrich) and 300 μg/mL DNase I (Roche Diagnostics, Machelen, Belgium), dissolved in 3 mL Roswell Park Memorial Institute medium (RPMI; Gibco), in a shaking heated bath (37°C). Obtained cell suspensions were filtered, red blood cells (RBCs) were removed by means of RBC Lysis Buffer (composed of 155 mM NH_4_Cl, 12 mM NaHCO_3_ and 0.1 mM EDTA) and cells were stained with appropriate antibodies for flow cytometry. During sacrification, mice were weighed, whole-liver weights were recorded, and the number of hepatic lesions was macroscopically evaluated. Large tumoral lesions were removed from the pieces of liver used for downstream analysis.

### Flow cytometry

Cells were prestained with a 1:100 dilution of Zombie Aqua (Fixable Viability Dye; BioLegend, London, UK) for 20 minutes at 4°C in the dark. After 10 minutes, an equal volume of a 1:100 dilution of TruStain FcX PLUS (anti-mouse CD16/32 antibody) and True-Stain Monocyte Blocker (BioLegend) was added. After a washing step, cells were stained with CD3e-PerCP-Cy5.5, CD19-PerCP-Cy5.5 (eBioscience, Thermo Fisher Scientific), NK1.1-PerCP-Cy5.5, CD103-PerCP-Cy5.5, F4/80-FITC, Ly6G-BV785, Ly6C-BV650 (BioLegend), SiglecF-PerCP-Cy5.5, CD45-APC-Cy7, CD11b-PE-Cy7 and Tim4-PE (BD Biosciences, Erembodegem, Belgium) for 20 minutes at 4°C in the dark. Cells were analyzed with a BD FACSAria Fusion flow cytometer (BD Biosciences) and FlowJo software (FlowJo LLC, BD Biosciences), and gated first as live CD45^+^ single cells. Subsequently, CD3e^+^, CD19^+^, NK1.1^+^, CD103^+^ and SiglecF^+^ cells were eliminated, and CD11b^+^Ly6C^+^Ly6G^-^ monocytes, CD11b^+^Ly6C^-^F4/80^+^Tim4^+^ Kupffer cells (KCs) and CD11b^+^Ly6C^-^F4/80^+^Tim4^-^ monocyte-derived macrophages (MoMfs) were gated.

### RT-qPCR

RNA was extracted from 20 mg of frozen liver tissue preserved in RNAlater, according to the instruction manual of the Aurum Total RNA Mini Kit (Bio-Rad Laboratories, Temse, Belgium), and measured for purity and quantity by spectrophotometry (NanoDrop; Thermo Fisher Scientific). cDNA was obtained from one microgram of RNA by reverse transcription using the SensiFAST cDNA Synthesis Kit (Bioline, London, UK) according to the manufacturer’s guidelines. Diluted cDNA (1:10) was subjected to 45 cycles of quantitative PCR amplification using SYBR Green mix (SensiMix; Bioline) and 2 μM of each primer (Biolegio, Nijmegen, The Netherlands). A 2-step program was run on a LightCycler 480 (Roche Diagnostics). Melting curve analysis confirmed primer specificities. All reactions were run in duplicate and normalized to reference genes that showed stable expression in all samples: hydroxymethylbilane synthase (HMBS), hypoxanthine-guanine phosphoribosyltransferase (HPRT) and succinate dehydrogenase complex flavoprotein subunit A (SDHA). The PCR efficiency of each primer pair was calculated using a standard curve of reference cDNA. Amplification efficiency was determined using the formula 10^-1/slope^-1. The sequences of the used primer pairs are listed in Supplementary Table 1.

### Multiplex analyses of pro-inflammatory cytokines/chemokines

Snap-frozen liver tissue was thawed in 1 mg/mL protease inhibitor cocktail (cOmplete, Mini, EDTA-free Protease Inhibitor Cocktail; Roche Diagnostics), 1 vol% phosphatase inhibitor cocktail 2 (Sigma-Aldrich) and 1 vol% phosphatase inhibitor cocktail 3 (Sigma-Aldrich) in PBS, lysed by sonication and centrifuged (15 minutes, 15,000 rpm, 4°C). The supernatant was stored at –80°C until further analysis. Total protein concentrations were measured using the Pierce BCA Protein Assay Kit (Thermo Fisher Scientific) according to the manufacturer’s guidelines. Protein levels of TNFα, IL-6, IFN-γ, CCL2 and CCL5 were determined by a bead-based Bio-Plex multiplex immunoassay (Bio-Rad Laboratories) according to the manufacturer's guidelines.

### Histology

Liver samples were fixed in 4% phosphate-buffered formaldehyde solution, dehydrated, embedded in paraffin, and sectioned (5 μm sections). Liver sections were stained with Sirius Red (Sigma-Aldrich). The extent of fibrosis was visualized using an Olympus BX41 microscope (Olympus, Antwerp, Belgium) and Cell^D software (Olympus), and scored using the Metavir scoring system. The scoring was carried out by two independent researchers, who were blinded to the study samples.

### Statistical analysis

Statistical analysis was performed using GraphPad Prism 6 (GraphPad Software, San Diego, California, USA). Normality was evaluated with the D’Agostino-Pearson omnibus test. Outliers were identified with the ROUT method, and excluded from the datasets. The maximum allowed false discovery rate was set to 1%. Normally distributed data were analyzed with the Student’s *t*-test or one-way analysis of variance (ANOVA) corrected with the Holm-Sidak test. Non-normally distributed data were analyzed using the Mann–Whitney *U* test or the Kruskal-Wallis test corrected with Dunn’s multiple comparisons test. RT-qPCR data are expressed as log2-transformed mean ± SD relative to the log2-transformed mean of the control. Other measurements are expressed as median or mean ± SD. Two-tailed *p*-values < 0.05 were considered statistically significant (^*^ *p* < 0.05, ^**^ *p* < 0.01, ^***^ *p* < 0.001 and ^****^ *p* < 0.0001).

## SUPPLEMENTARY MATERIALS



## Abbreviations

α-SMA: α-smooth muscle actin
AFP: α-fetoprotein
AKT: protein kinase B
ALD: alcoholic liver disease
ANOVA: analysis of variance
ASO: antisense oligonucleotide
CBP: CREB-binding protein
CCL: C-C motif chemokine ligand
CCR: C-C motif chemokine receptor
CTLA-4: cytotoxic T-lymphocyte-associated protein 4
CXCL: C-X-C motif chemokine ligand
DEN: N:N-diethylnitrous amide
ECM: extracellular matrix
EPO: erythropoietin
FIH: factor inhibiting HIF
GAPDH: glyceraldehyde 3-phosphate dehydrogenase
GLUT1: glucose transporter 1
GPC3: glypican-3
HCC: hepatocellular carcinoma
HDAC: histone deacetylase
HIF: hypoxia-inducible factor
HMBS: hydroxymethylbilane synthase
HPRT: hypoxanthine-guanine phosphoribosyltransferase
HRE: hypoxia response element
Hsp90: heat shock protein 90
IFN: interferon
IGF-2: insulin-like growth factor 2
IL: interleukin
iNOS: inducible nitric oxide synthase
IRE: iron-responsive element
IRP1: iron-regulatory protein 1
KC: Kupffer cell
LOX: lysyl oxidase
MMP: matrix metalloproteinase
MoMf: monocyte-derived macrophage
mRNA: messenger ribonucleic acid
mTOR: mammalian target of rapamycin
NaCl: sodium chloride
NAFLD: non-alcoholic fatty liver disease
PBS: phosphate-buffered saline
PD-1: programmed cell death protein 1
PDGF: platelet-derived growth factor
PGK1: phosphoglycerate kinase 1
PHD: prolyl hydroxylase domain-containing protein
PI3K: phosphoinositide 3-kinase
pVHL: von Hippel-Lindau tumor suppressor protein
RBC: red blood cell
RPMI: Roswell Park Memorial Institute medium
RT-qPCR: quantitative reverse transcription-polymerase chain reaction
SDHA: succinate dehydrogenase complex flavoprotein subunit A
TGF: transforming growth factor
TIMP: tissue inhibitor of metalloproteinase
TME: tumor microenvironment
TNF: tumor necrosis factor
VCAM: vascular cell adhesion molecule
VEGF: vascular endothelial growth factor
VEGFR: vascular endothelial growth factor receptor
