# Supplementary material for: Effect of isoform-specific HIF-1α and HIF-2α antisense oligonucleotides on tumorigenesis, inflammation and fibrosis in a hepatocellular carcinoma mouse model
Source: Oncotarget. 2020 Dec 1;11(48):4504–20. doi: 10.18632/oncotarget.27830 (PMC7721613; doi:10.18632/oncotarget.27830)
Supplement: Supplementary file 1 [file oncotarget-11-4504-s001.pdf]

Effect of isoform-specific HIF-1α and HIF-2α antisense oligonucleotides on tumorigenesis, inflammation and fibrosis in a hepatocellular carcinoma mouse model

SUPPLEMENTARY MATERIALS

Supplementary Table 1: Primer pairs used for RT-qPCR

Gene	Full name	Primer sequences (5′–3′)	E (%)
Acta2	aortic smooth muscle actin alpha 2	Fwd: GTCCCAGACATCAGGGAGTAA<br>Rev: TCGGATACTTCAGCGTCAGGA	103
Afp	alpha fetoprotein	Fwd: AGCTTCCACGTTAGATTCCTCC<br>Rev: ACAAACTGGGTAAAGGTGATGG	111
Ccl2	C-C motif chemokine ligand 2	Fwd: TTAAAAACCTGGATCGGAACCAA<br>Rev: GCATTAGCTTCAGATTTACGGGT	101
Ccr2	C-C motif chemokine receptor 2	Fwd: ATCCACGGCATACTATCAACATC<br>Rev: CAAGGCTCACCATCATCGTAG	98
Cxcl2	C-X-C motif chemokine ligand 2	Fwd: GCGCCCAGACAGAAGTCATAG<br>Rev: AGCCTTGCCTTTGTTCAGTATC	89
Epas1	endothelial PAS domain protein 1<br>(= hypoxia inducible factor 2 alpha)	Fwd: GTGACATGATCTTTCTGTCGGAA<br>Rev: CGCAAGGATGAGTGAAGTCAAA	94
Gpc3	glypican 3	Fwd: GGTCACGTCTTGCTCCTCG<br>Rev: TCGACAGCCTCTTTCCCAGTCA	103
Hif1a	hypoxia inducible factor 1 alpha	Fwd: ACTCCTAACTTTTCCCAGCCT<br>Rev: TGACCATATCGCTATCCACATCA	96
Hif3a	hypoxia inducible factor 3 alpha	Fwd: GAAGTTCACATACTGCGACGA<br>Rev: GTCCAAAGCGTGGATGTATTCAT	87
Hmbs	hydroxymethylbilane synthase	Fwd: AAGGGCTTTTCTGAGGCACC<br>Rev: AGTTGCCCATCTTTCATCACTG	95
Hprt	hypoxanthine-guanine phosphoribosyltransferase	Fwd: GTTAAGCAGTACAGCCCCAAA<br>Rev: AGGGCATATCCAACAACAAACTT	96
Il6	interleukin 6	Fwd: GCTGGTGACAACCACGGCCT<br>Rev: AGCCTCCGACTTGTGAAGTGGT	92
Mmp2	matrix metalloproteinase 2	Fwd: CAAGTTCCCCGGCGATGTC<br>Rev: TTCTGGTCAAGGTCACCTGTC	106
Mmp10	matrix metalloproteinase 10	Fwd: GAGCCACTAGCCATCCTGG<br>Rev: CTGAGCAAGATCCATGCTTGG	111
Mmp14	matrix metalloproteinase 14	Fwd: GCTTCCGAGAAGTGCCCTATG<br>Rev: TCCTTCACCATCAAAGGGTGTA	104
Mmp16	matrix metalloproteinase 16	Fwd: AGAAGGTTGGATTTCGTGCAT<br>Rev: TCCGCAGACTGTAGCACATAA	93
Nos2	inducible nitric oxide synthase 2	Fwd: GGCAGCCTGTGAGACCTTTG<br>Rev: GCATTGGAAGTGAAGCGTTC	99

Sdha	succinate dehydrogenase complex flavoprotein subunit A	Fwd: CTTGAATGAGGCTGACTGTG<br>Rev: ATCACATAAGCTGGTCCTGT	103
Tgfb1	transforming growth factor beta	Fwd: TGAGCGTCACTGGAGTTGTACGG<br>Rev: GGTTCATGTCATGGATGGTGC	95
Timp1	tissue inhibitor of metalloproteinase 1	Fwd: CTTGGTTCCCTGGCGTACTC<br>Rev: ACCTGATCCGTCCACAAACAG	100
Tnf	tumor necrosis factor alpha	Fwd: CATCTTCTCAAAATTCGAGTGACAA<br>Rev: TGGGAGTAGACAAGGTACAACCC	106
Vcam1	vascular cell adhesion molecule 1	Fwd: CCTTGTGGAGGGATGTACAGA<br>Rev: TGCCGAGCTAAATTACACATTG	104

Target species is *Mus musculus* (house mouse) for all primers. Fwd = forward, Rev = reverse, E = amplification efficiency.
